# Detection of ABCC1 expression in classical Hodgkin lymphoma is associated with increased risk of treatment failure using standard chemotherapy protocols

**DOI:** 10.1186/1756-8722-5-47

**Published:** 2012-08-07

**Authors:** Wesley Greaves, Lianchun Xiao, Beatriz Sanchez-Espiridion, Kranthi Kunkalla, Kunal S Dave, Cynthia S Liang, Rajesh R Singh, Anas Younes, L Jeffrey Medeiros, Francisco Vega

**Affiliations:** 1 Department of Hematopathology, Unit 72, The University of Texas MD Anderson Cancer Center, 1515 Holcombe Boulevard, Houston, TX, 77030, USA; 2 Biostatistics, The University of Texas MD Anderson Cancer Center, Houston, TX, 77030, USA; 3 Lymphoma & Myeloma, The University of Texas MD Anderson Cancer Center, Houston, TX, 77030, USA

**Keywords:** Classical Hodgkin lymphoma, ABCC1, ATP binding cassettes, Immunohistochemistry

## Abstract

**Background:**

The mechanisms responsible for chemoresistance in patients with refractory classical Hodgkin lymphoma (CHL) are unknown. ATP-binding cassette (ABC) transporters confer multidrug resistance in various cancers and *ABCC1* overexpression has been shown to contribute to drug resistance in the CHL cell line, KMH2.

**Findings:**

We analyzed for expression of five ABC transporters ABCB1, ABCC1, ABCC2, ABCC3 and ABCG2 using immunohistochemistry in 103 pre-treatment tumor specimens obtained from patients with CHL. All patients received first-line standard chemotherapy with doxorubicin (Adriamycin®), bleomycin, vinblastine, and dacarbazine (ABVD) or equivalent regimens. ABCC1 was expressed in Hodgkin and Reed-Sternberg (HRS) cells in 16 of 82 cases (19.5%) and ABCG2 was expressed by HRS cells in 25 of 77 cases (32.5%). All tumors were negative for ABCB1, ABCC2 and ABCC3. ABCC1 expression was associated with refractory disease (p = 0.01) and was marginally associated with poorer failure-free survival (p = 0.06). Multivariate analysis after adjusting for hemoglobin and albumin levels and age showed that patients with CHL with HRS cells positive for ABCC1 had a higher risk of not responding to treatment (HR = 2.84, 95%, CI: 1.12-7.19 p = 0.028).

**Conclusions:**

Expression of ABCC1 by HRS cells in CHL patients predicts a higher risk of treatment failure and is marginally associated with poorer failure-free survival using standard frontline chemotherapy regimens.

## Background

Classical Hodgkin lymphoma (CHL) is largely a curable disease using the widely accepted current standard first-line chemotherapy regimen of doxorubicin (Adriamycin®), bleomycin, vinblastine, and dacarbazine (ABVD) or equivalent regimens, with or without consolidation radiotherapy [1]. However, approximately 20% of patients with CHL do not respond following first-line therapy, or relapse quickly, and require additional treatment with salvage chemotherapy with or without stem cell transplantation [1,2]. A drawback to the currently used treatment modalities is their association with potentially life-threatening toxicities. In addition, patients cured of CHL have an increased lifetime relative risk of death from non CHL-related causes, presumably attributable, at least in part, to therapy [3]. Thus, investigators continue to actively pursue novel prognostic biomarkers and therapeutic options in CHL patients with the goals of maintaining or improving survival rates as well as minimizing adverse side effects in patients with favorable prognosis [2]. Recently, a number of biomarkers expressed by Hodgkin and Reed-Sternberg (HRS) cells as assessed in tissue samples have been proposed as being useful for predicting prognosis in CHL patients [4]. These molecules include matrix metalloproteinase 11 (MMP11), CD20, Bcl2, MAL, HLA class II and Ki67, as well as cells within the CHL microenvironment, such as tumor-associated macrophages or subsets of tumor-infiltrating lymphocytes, including FOXP3+ regulatory T cells (Tregs) and granzyme B + T/NK cells [5-10].

The development of chemotherapy resistance by cancer cells is multifactorial [11]. ATP binding cassette (ABC) transporters comprise a ubiquitous family of transmembrane proteins that play a physiologic role in the transport of substrates across cytoplasmic membranes. ABC transporters also play a role in multidrug resistance (MDR) in multiple tumor types by using ATP as an energy source to actively expel drug substrates from the tumor cell cytoplasm into the extracellular space [12]. Expression of ABC transporters has been shown to correlate with response to therapy and prognosis in several hematological malignancies including acute myeloid leukemia and diffuse large B-cell lymphoma [13-15]. Although the clinical impact of ABC transporters in CHL has not been reported, several drugs used to treat CHL are known substrates of various ABC transporters [11,16], including doxorubicin (a substrate for ABCB1, ABCC1, ABCC2, ABCC3, ABCG2), vinblastine (a substrate for ABCB1 and ABCC1) and vincristine (a substrate for ABCC1).

Steidl et al. recently showed overexpression of the ABC transporter, ABCC1 (also known as multidrug resistance protein 1 - MRP1) in the therapy-resistant CHL-derived cell line, KMH2 [17]. They further showed that increased sensitivity of KMH2 cells to Adriamycin® toxicity by siRNA silencing of *ABCC1*. Prompted by this finding, we assessed for expression of five ABC transporters, ABCG2, ABCB1, ABCC1, ABCC2, and ABCC3, in untreated CHL tumor specimens. We also investigated the potential prognostic value of expression of these ABC transporters in CHL.

## Design and methods

The overall clinical and pathologic features of the study group are summarized in Table 1. The group included 103 patients with CHL who were seen at our hospital and treated with standard front-line chemotherapy using ABVD (36 patients) or equivalent regimens including CVPP/ABDIC (cyclophosphamide, vinblastine, procarbazine, and prednisone/Adriamycin®, bleomycin, dacarbazine, lomustine and prednisone) (20 patients), MOPP/ABVD (mechlorethamine, vincristine, prednisone, procarbazine/Adriamycin®, bleomycin, vinblastine, dacarbazine) (3 patients) or NOVP (Novantrone®, vincristine, vinblastine, and prednisone) (44 patients) with and without radiotherapy. Additionally, 10 patients underwent allogeneic stem cell transplantation as salvage therapy. We analyzed for expression of five ABC transporters - ABCG2, ABCB1, ABCC1, ABCC2, and ABCC3 - in pre-treatment samples of CHL using (see Table 2). immunohistochemical methods and tissue microarrays (TMA). Seven TMAs were constructed using triplicate cores prepared from routinely processed paraffin-embedded tissue specimens as described previously [18]. Additionally, we were able to retrieve tissue blocks and use routine histologic sections to analyze ABCC1 and ABCG2 expression in 13 and 5 CHL tumors, respectively, that suffered tissue loss on the TMAs. This work was performed under an approved IRB protocol in our institution. For each marker, a tumor was considered positive when HRS cells were positive. For these proteins expression was all or none. In other words, in positive cases virtually all HRS cells were positive.

**Table 1:** Selected demographic and histologic features of 103 CHL patients

**Parameter**	**n (%)**
Gender	
Male	59 (57.3%)
Female	44 (42.7%)
Mean age	36 years (range: 13–85)
Age ≥ 45 years	28 (27%)
Ann Arbor Stage	
I	9 (8.7%)
II	48 (46.6%)
III	26 (25.2%)
IV	20 (19.4%)
IPS	
< 3	83 (80.6%)
≥ 3	20 (19.4%)
Radiotherapy	
No	21 (22.6%)
Yes	72 (77.4%)
Chemotherapy	
ABVD	34 (33%)
ABVD + rituximab	2 (1.94%)
CVPP/ABDIC	20 (19.4%)
MOPP/ABVD	3 (2.9%)
NOVP	44 (42.7%)
CHL Histologic Subtype:	
Nodular sclerosis	75 (72.8%)
Mixed cellularity	22 (21.3%)
Lymphocyte rich	3 (2.9%)
Lymphocyte depleted	3 (2.9%)

**Table 2:** Antibodies used for immunohistochemistry

**Antibody Common Name**	**Systematic Name**	**Clone**	**Manufacturer**	**Antibody Conc.**	**Normal Tissue Control**
ABCG2	MXR, BCRP, ABC-P	Mouse monoclonal BXP-21	Santa Cruz Biotechnology Inc. Santa Cruz, CA	1:40	Placenta
MDR1	ABCB1, PGP	Mouse monoclonal G-1	Santa Cruz Biotechnology Inc. Santa Cruz, CA	1:100	Liver
MRP1	ABCC1	Mouse monoclonal QCRL-1	Santa Cruz Biotechnology Inc. Santa Cruz, CA	1:50	Stomach
MRP2	ABCC2	Mouse monoclonal M2 III-6	Abcam Inc. Cambridge MA	1:50	Liver
MRP3	ABCC3	Mouse monoclonal DTX1	Abcam Inc. Cambridge MA	1:50	Liver

Fisher’s exact test was used to evaluate the association of clinical response with categorical variables. The Kaplan-Meier method and log rank test were used for survival analysis. The following variables were evaluated in univariate analysis: disease stage (IV vs. I/II/III), chemotherapy (ABVD, CVP or NOVP), radiation therapy (yes and no), bone marrow metastasis (positive and negative), serum albumin (< and > 40 g/L), WBC (< and ≥ 15,000 per mm^3^), hemoglobin (< or > 105 g/L), lymphocytes (< and ≥ 600 per mm^3^ or < and ≥ 8% of WBC), gender, International Prognostic Score (IPS) (< and ≥ 3), and age (< and ≥ 45 years). Multivariate Cox proportional hazards models including variables with p value < 0.15 in univariate analysis were fitted to evaluate the association of survival with demographic and clinical factors. Variables with p values < 0.05 were considered statistically significant. S plus software 8.04 (TIBCO software Inc., Palo Alto, CA) and SAS software (SAS Institute Inc., Cary, NC) were used for statistical analysis.

## Results and discussion

**Figure 1:**
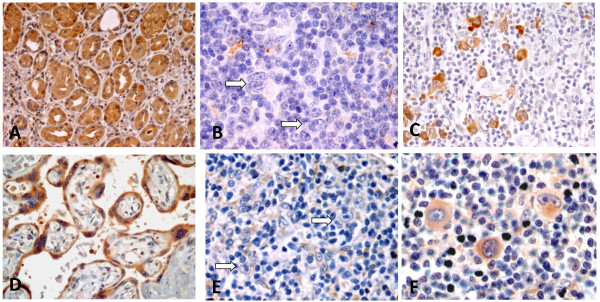
**Immunohistochemical expression of ABC proteins in positive controls and CHL tumors.A**. ABCC1 is expressed by gastric glands (positive control). **B**. CHL with negative expression of ABCC1 by HRS cells; scattered histiocytes are weakly positive. **C**. CHL with HRS cells positive for cytoplasmic expression of ABCC1. **D**. Placenta with ABCG2 expression in trophoblastic cells (positive control). **E**. CHL with HRS cells negative for ABCG2; endothelial cells and scattered inflammatory cells are positive. **F**. CHL with HRS cells positive for cytoplasmic expression of ABCG2.

**Figure 2:**
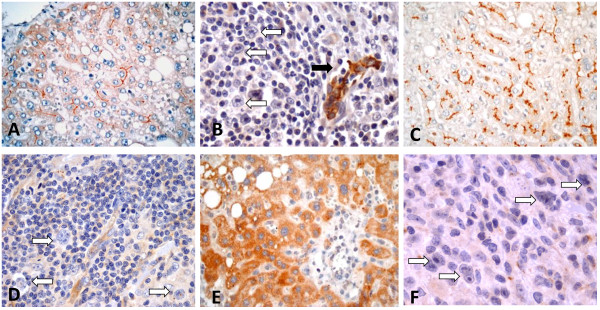
**Immunohistochemical expression of ABC proteins in positive controls and call tumors.** A. Canalicular staining pattern of MDR1 in liver (positive control). **B**. MDR1 is not expressed in the HRS cells of CHL (white arrows); endothelial cells are positive (black arrow). **C**. Canalicular staining pattern of ABCC2 in liver (positive control). **D**. HRS cells are negative for ABCC2; endothelial cells and scattered lymphocytes are positive. **E**. Hepatocytes show cytoplasmic expression of ABCC3 (positive control). **F**. HRS cells are negative for ABCC3.

We tested for expression of five ABC transporters in untreated tumor specimens of CHL. These transporters use as substrates chemotherapeutic agents commonly used to treat CHL patients including Adriamycin®, vincristine, vinblastine, and mitoxantrone, among others [11]. ABCG2 and ABCC1 were expressed by HRS cells in a subset of CHL tumors (Figure 1). Sixteen of 82 (19.5%) CHL were positive for ABCC1 and 25 of 77 (32.5%) CHL were positive for ABCG2 (a subset of tissue cores was variably lost on the TMAs). Both ABCC1 and ABCG2 showed cytoplasmic expression in all HRS cells (Figures 1C and F). There was no substantial difference in the intensity of expression of ABCC1 or ABCG2 by HRS cells. Variable, non-specific staining for ABCC1 and ABCG2 was also observed inconsistently in a small subset of background inflammatory cells, including plasma cells, lymphocytes, eosinophils and histiocytes, in both HRS-positive and HRS-negative cases. There was no expression of ABCB1, ABCC2 and ABCC3 by HRS cells in any case analyzed (Figures 2B, D and E). Consistent expression of both ABCC1 and ABCG2 in endothelial cells was used as an internal positive control for immunohistochemical staining (see Figures 2B and D).

**Figure 3:**
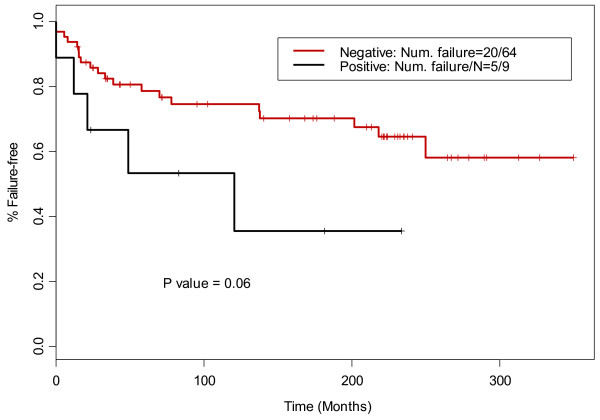
ABCC1 expression was marginally associated with failure-free survival (p = 0.06).

We sought to determine if there was an association between expression of either ABCC1 or ABCG2 and clinical endpoints, such as response to treatment (refractory disease vs non-refractory disease), overall survival (OS), and failure free survival (FFS). Admittedly, the numbers are relatively small hampering this analysis. In this study FFS was defined as lack of disease progression, recurrence or death. Refractory disease was defined as patients with only a partial response to therapy, or recurrence within the first 18 months of initial therapy [19,20]. The log rank test showed that ABCC1 expression was marginally associated with FFS: 19 of 66 ABCC1 negative patients and 7 of the ABCC1 positive patients experienced treatment failure. The estimated 5-year FFS probabilities were 80.7% (95% CI:71.4% -91.2%) for ABCC1 negative group and 68.8% (95% CI:49.4%-95.7%) for the ABCC1 positive group, respectively (p = 0.06, Figure 3). Multivariate analysis after adjusting for the effects of age, hemoglobin level, and albumin level suggested that ABCC1 expression was an independent prognostic marker for FFS. Patients with ABCC1 expression had a higher risk of treatment failure than patients without ABCC1 expression (HR = 2.88, 95% CI: 1.18-7.01, p = 0.02, Table 3). Fisher’s exact test suggested that ABCC1 expression was also associated with initial response to treatment (primary refractory *vs* non-primary refractory). Six of 16 patients (37.5%) with ABCC1 expression *versus* 6 of 66 patients (9.1%) without ABCC1 expression were primary refractory (p = 0.01). This finding supports the results of Steidl and colleagues in the KMH2 cell line [17] and suggests that expression of ABCC1 may contribute to primary drug resistance in CHL. Three of 16 patients with ABCC1 positive tumors and 11 of 66 patients with ABCC1 negative tumors died, no significant difference was detected in OS between the ABCC1 positive and negative groups (p = 0.74). ABCC1 expression was not significantly associated with other clinical parameters (Table 4). Fisher’s exact test was also used to compare the patient characteristics between ABCC1 known and ABCC1 unknown groups (Additional file 1: Table S1). More patients 45 years of age or older had ABCC1 measurements (17/28, 60.7%) (p value =0.0036). A majority of patients who received CVPP treatment had ABCC1 measurements (18/20, 90%) (p value = 0.049). No other significant difference was detected.

**Table 3:** Multivariate analysis to evaluate the association between FFS and ABCC1

		**HR (95% CI)**	**P value**
ABCC1	Positive vs. negative	2.84 (1.12, 7.19)	0.028
Albumin	<4 vs. > 4	1.59 (0.70, 3.63)	0.27
Age	> = 45 vs. <45	2.14 (1.53, 0.13)	0.13

*Please note: HB (<10.5 vs. > 10.5) was included in the model as a stratification factor since the proportional hazards assumption for it was not held.

**Table 4:** Fisher’s exact test to evaluate the association between ABCC1 and other clinical factors

**Covariate**	**Score**	**ABCC1 Negative**	**ABCC1 Positive**	**Fisher's ExactTest (2-Tail)**
ABCG2	Negative	39 (79.6%)	10 (20.4%)	.5227
	Positive	22 (88%)	3 (12%)	
Chemotherapy	ABVD (R-ABVD & MOPP/ABVD)	20 (76.9%)	6 (23.1%)	.8762
	CVPP/ABDIC	15 (83.3%)	3 (16.7%)	
	NOVP	31 (81.6%)	7 (18.4%)	
Radiotherapy	No	17 (89.5%)	2 (10.5%)	.3329
	Yes	45 (77.6%)	13 (22.4%)	
Bone marrow disease	No	62 (79.5%)	16 (20.5%)	1.000
	Yes	3 (100%)	0 (0%)	
Stage IV disease	No	53 (80.3%)	13 (19.7%)	1.000
	Yes	13 (81.3%)	3 (18.8%)	
Hemoglobin	≥105 g/l	58 (79.5%)	15 (20.5%)	.6811
	< 105 g/l	8 (88.9%)	1 (11.1%)	
Albumin	≥ 40 g/l	35 (79.5%)	9 (20.5%)	1.000
	< 40 g/l	23 (82.1%)	5 (17.9%)	
WBC	<15,000 per mm^3^	62 (79.5%)	16 (20.5%)	.5814
	≥15,000 per mm^3^	4 (100%)	0 (0%)	
Lymphocytes	< 600 per mm^3^	52 (80%)	13 (20%)	1.000
	≥ 600 per mm^3^	11 (78.6%)	3 (21.4%)	
Age ≥45	< 45 years	51 (78.5%)	14 (21.5%)	.5028
	≥ 45 years	15 (88.2%)	2 (11.8%)	
Sex	Female	26 (76.5%)	8 (23.5%)	.5731
	Male	40 (83.3%)	8 (16.7%)	
IPS	<3	51 (77.3%)	15 (22.7%)	.2811
	≥3	14 (93.3%)	1 (6.7%)	

Expression of ABCG2 by HRS cells was not significantly associated with OS, FFS or initial response to treatment. The lack of association of ABCG2 expression with treatment refractoriness, in contrast to ABCC1, is not fully explained, and relatively little is known about the differential substrate profiles of these two proteins. However, some authors have shown that certain drugs that are poor ABCC1 substrates, such as mitoxantrone (a type 2 topoisomerase inhibitor), are associated with overexpression of ABCG2 *in vitro *[11,21], and such differences may have played a role in the discordant impact of these two proteins on therapy resistance in this patient cohort.

## Conclusions

In summary, ABCC1 and ABCG2 are expressed by HRS cells in a subset of CHL tumors. Univariate and multivariate analyses showed that expression of ABCC1 by HRS cells is associated with an increased risk of tumor progression, treatment resistance or death in CHL patients. Our findings corroborate those published by Steidl and colleagues [17] in the KMH2 cell line and provide evidence that expression of ABCC1 may be useful as an indicator of poorer FFS or failure to respond to therapy in CHL patients who are treated with standard regimens. Additionally, ABCC1 may serve as a potential target for therapeutic intervention by increasing susceptibility to chemotherapy.

## Abbreviations

CHL: Classical Hodgkin lymphoma; ABVD: Adriamycin, bleomycin, vinblastine, and dacarbazine; HRS: Hodgkin Reed-Sternberg cells; MMP11: Matrix metalloproteinase 11; ABC: ATP binding cassette; MDR: Multidrug resistance; CVPP: Cyclophosphamide, vinblastine, procarbazine, and prednisone; NOVP: Novantrone, vincristine, vinblastine, and prednisone; TMA: Tissue microarrays; WBC: White blood count; IPS: International prognostic index; FFS: Failure free survival; OS: Overall survival.

## Competing interest

The authors indicated no potential conflicts of interest.

## Authors’ contributions

WG carried out data analysis and interpretation and wrote the manuscript. LX and BSE carried out data analysis and interpretation and performed statistical analysis. AY participated in the provision of clinical data and patient samples. KSD and CSL participated constructing the tissue microarrays. KK performed the immunohistochemical studies. LJM participated in providing of patient samples, data analysis, and the writing of the manuscript. FV conceived of the study, performed data analysis and interpretation, and wrote the manuscript. Final approval of the manuscript: All the co-authors.

## Supplementary Material

Additional file 1 Table S1. Fisher’s exact test to compare clinical factors between ABCC1 unknown and ABCC1 known groups. Click here for file
