# Supplementary material for: Detection of ABCC1 expression in classical Hodgkin lymphoma is associated with increased risk of treatment failure using standard chemotherapy protocols
Source: J Hematol Oncol. 2012 Aug 7;5:47. doi: 10.1186/1756-8722-5-47 (PMC3470996; doi:10.1186/1756-8722-5-47)
Supplement: Additional file 1 — Table S1. Fisher’s exact test to compare clinical factors between ABCC1 unknown and ABCC1 known groups. [file 1756-8722-5-47-S1.doc]

Table: Fisher’s exact test to compare clinical factors between ABCC1 unknown and ABCC1 known groups

| **Variable** | **levels** | ABCC1 unknown | ABCC1 known | **P value** |
| --- | --- | --- | --- | --- |
| ABCG2 | Negative | 3(5.8%) | 49(94.2%) | .5468 |
|  | Positive | 0(0%) | 25(100%) | . |
| Chemotherapy | ABVD | 13(33.3%) | 26(66.7%) | .0494 |
|  | CVPP | 2(10%) | 18(90%) | . |
|  | NOVP | 6(13.6%) | 38(86.4%) | . |
| Radiotherapy | No | 2(9.5%) | 19(90.5%) | .5109 |
|  | Yes | 14(19.4%) | 58(80.6%) | . |
| Bone marrow disease | No | 18(18.8%) | 78(81.3%) | .2564 |
|  | Yes | 2(40%) | 3(60%) | . |
| STAGE_IV disease | No | 16(19.5%) | 66(80.5%) | 1.000 |
|  | Yes | 4(20%) | 16(80%) | . |
| Hemoglobin | ≥105g/l | 16(18%) | 73(82%) | .2776 |
|  | < 105g/l | 4(30.8%) | 9(69.2%) | . |
| Albumin | ≥ 40g/l | 10(18.5%) | 44(81.5%) | .5563 |
|  | < 40g/l | 4(12.5%) | 28(87.5%) | . |
| WBC | <15,000 per mm^3^ | 19(19.6%) | 78(80.4%) | 1.000 |
|  | ≥15,000 per mm^3^ | 1(20%) | 4(80%) | . |
| Lymphocytes | < 600 per mm^3^ | 17(20.7%) | 65(79.3%) | .7302 |
|  | ≥ 600 per mm^3^ | 2(12.5%) | 14(87.5%) | . |
| AGE | < 45 years | 10(13.3%) | 65(86.7%) | .0036 |
|  | ≥ 45 years | 11(39.3%) | 17(60.7%) | . |
| SEX | Female | 10(22.7%) | 34(77.3%) | .6109 |
|  | Male | 11(18.6%) | 48(81.4%) | . |
| IPS | <3 | 13(16.5%) | 66(83.5%) | .3529 |
|  | >=3 | 5(25%) | 15(75%) | . |
